# A fast measure of spatial separation for release from masking and its relation to intelligibility

**DOI:** 10.1121/10.0035840

**Published:** 2025-02-13

**Authors:** Z. Ellen Peng, Victoria Sweeney

**Affiliations:** Functional Hearing Laboratory, Boys Town National Research Hospital, Omaha, Nebraska 68131, USA Ellen.Peng@boystown.org; Victoria.sweeney@boystown.org

## Abstract

This study validates a fast measure for spatial release from masking—minimum angular separation (MAS), the smallest spatial separation between a target and two-talker maskers to improve speech intelligibility by 20%. Three psychophysical methods to estimate MAS were compared, including the constant stimuli, adaptive staircase, and progressive tracking, which revealed no significant difference in the estimated threshold on the group level with bootstrapping. Results suggest that the MAS measurement can be expedited using the progressive tracking method without compromising robustness in the threshold estimation. The non-linear relationship between target-masker spatial separation, signal-to-noise ratio, and accuracy is explored.

## 1. Introduction

Spatial unmasking refers to a benefit in which listeners can use a spatial separation between the target speech and interfering maskers to improve identification accuracy (Brungart *et al.*, 2001). It is an important measure to assess spatial hearing abilities in listeners in the context of speech understanding in complex listening environments. It has been widely used in spatial hearing studies to assess the impact of hearing status and device use in various listener populations (Cubick *et al.*, 2018; Edmonds and Culling, 2005a; Hawley *et al.*, 2004; Srinivasan *et al.*, 2016), to understand auditory development across the lifespan (Corbin *et al.*, 2017; Griffin *et al.*, 2019; Litovsky, 2005; Peng *et al.*, 2021; Srinivasan *et al.*, 2016; Van Deun *et al.*, 2010), and to investigate the effects of acoustic degradation on spatial cues (Ahrens *et al.*, 2020; Marrone *et al.*, 2008; Peng *et al.*, 2021).

There are three parameters involved traditionally to quantify the amount of intelligibility gain as spatial unmasking: (1) spatial separation between the target and masker, (2) signal-to-noise ratio (SNR) between the target and masker, and (3) identification accuracy of the target. Two test conditions are typically compared to derive the amount of unmasking or benefit. Between the two conditions, one parameter is fixed (e.g., percent correct at 70%) and one parameter incurs a known change (e.g., target and masker co-located vs displaced at a known spatial separation such as 90°), allowing the third parameter to vary across conditions to be quantified as the amount of unmasking or benefit (e.g., SNR difference). Because allowing target identification accuracy to vary may result in floor or ceiling effect, most studies on spatial unmasking have been characterizing it as the change in SNR at a fixed accuracy, also known as the speech reception threshold (SRT).

More recently, several studies have adopted a procedure to measure spatial unmasking by the smallest spatial separation between the target and maskers instead of SRT change (Ahrens *et al.*, 2020; Ozmeral and Higgins, 2022; Peng and Litovsky, 2022). The minimum angular separation (MAS) is a threshold that measures the smallest amount of auditory spatial cues necessary from the target-masker separation. In the procedures by Ahrens *et al.* (2020) and Ozmeral and Higgins (2022), the fixed parameter was the target identification accuracy at 70% correct with a known change introduced to the SNR (e.g., reducing by 6 or 9 dB) between the two test conditions. In the procedure by Peng and Litovsky (2022), the fixed parameter was the SNR with a known change in target identification accuracy chosen at a 20% increase between conditions. Both procedures involve first measuring the listener's individual SRT when the target and maskers are co-located, then in the second step varying the angular separation adaptively to measure the MAS threshold.

There are two major benefits of measuring MAS with a fixed SNR across conditions. Listeners with typical hearing are generally sensitive to the spatial separation between the target and masker even though the separation is small (Srinivasan *et al.*, 2016), suggesting access to auditory spatial cues varies at different SNRs. When the type of acoustic degradation (e.g., reverberation) may deteriorate both speech cues and spatial cues, an MAS measured with fixed SNR can better disentangle reverberation's unique impact on speech intelligibility vs spatial unmasking. The second benefit of fixing SNR at individual listeners' co-located SRT is the enhanced ability to compare MAS across listener groups who may use different listening strategies at varying SNRs. For instance, positive SNR is a strong perceptual cue for target identification (Swaminathan *et al.*, 2015). Evaluating spatial unmasking across two conditions tested at SNRs above and below 0 dB may result in underestimating the unmasking benefit (Leibold *et al.*, 2020; Peng *et al.*, 2021). Moreover, children or individuals with hearing loss often require positive SNRs for speech-in-speech understanding with considerable individual differences, who may be unable to perform the task entirely after a fixed reduction of SNR. However, even children with bilateral cochlear implants can perform spatial unmasking using binaural or monaural auditory spatial cues at their individual SRTs (Peng and Litovsky, 2022).

Using the procedure to measure MAS with a fixed SNR (found at the listener's 50% accuracy with co-located target and maskers), the present study investigated several factors and their impact on the MAS thresholds. First, we compared how psychophysics methods affect the MAS thresholds to explore options to reduce assessment time. There are three major methods in psychoacoustic threshold estimation: The method of constant stimuli, adaptive staircase, and progressive tracking (Moore, 2012). The method of constant stimuli is more commonly used with the benefit of allowing the reconstruction of the entire psychometric function, followed by the adaptive staircase which can still be robust with reduced testing time by focusing testing more trials around the linear portion of the psychometric function where the threshold is typically estimated (Leek, 2001; Levitt, 1971). Progressive tracking is more commonly used in clinical speech tests for their efficiency in quickly estimating thresholds (e.g., Etymotic Research, 2001, 2005). There is recent validation work to suggest progressive tracking can be as robust as an adaptive staircase in estimating SRTs (Jakien *et al.*, 2017), which motivated our work in understanding its utility for MAS threshold estimation. We hypothesize that MAS measured using the method of constant stimuli has the best threshold estimate due to a large number of repetitions at each test angle and that other tracking methods, including adaptive staircase and progressive tracking, may result in under- or over-estimation of the MAS threshold, particularly for the progressive tracking methods with a low number of repetitions at each test angle.

Second, we assessed how MAS varied as measured from two target locations at 67.5° and 90°. For humans, interaural time difference (ITD) increases linearly for a sound moving from 0° to 90° azimuth, whereas interaural level difference (ILD) has a non-monotonic increase with a maximum at ∼67.5° (Algazi *et al.*, 2001). We measured angular separation from two target positions of 67.5° vs 90°, with the masker moving toward 0° azimuth, to allow us to explore the impact of natural ITD and ILD variation on MAS. We anticipated a larger MAS when the target was located at 90° azimuth, compared to when the target was located at 67.5°, due to diminishing ILD contrast between the target and masker when the masker moves from 90° to 67.5° before becoming available again toward azimuthal positions <67.5°. This also allowed us to examine any potential trade-off between ITD and ILD when used for MAS. Last, we explored the relations between spatial separation, SNR, and identification accuracy through additional MAS thresholds measured at different SNR levels.

## 2. Methods

### 2.1 Participants

Twenty-eight normal-hearing adults (11 males and 17 females) between 21 and 29 years of age (mean = 24.2 years, standard deviation = 2.4 years) participated in the study. All listeners passed a pure-tone hearing screen of 20 dB hearing level (HL) from 250 to 8000 Hz in octave band frequencies in each ear. Each participant received $20/h and completed the study in ∼1.5 h total over one or two visits.

### 2.2 Audio spatialization

Head-related transfer functions (HRTFs) recorded in the BTNRH anechoic chamber (ETS Lingren A100, Cedar Park, TX) were used to create spatial audio. A KEMAR head and torso simulator (GRAS Sound & Vibration, Holte, Denmark) was positioned in the center of a circular array of 64 loudspeakers (Orb Audio, New York, NY) on the horizontal plane spanning 360° with a spatial resolution of 5.625°. Outside the anechoic chamber, a desktop computer was used to play sine sweeps to the individual loudspeakers with a spectral range of 100 to 20 000 Hz at a sampling rate of 96 000 Hz through an external multi-channel Dante sound card and amplifier (Sonible d24; Sonible GmbH, Graz, Austria). Loudspeaker calibration was performed to ensure equal output from each speaker as measured by an omni-directional microphone in the center of the loudspeaker array. A pair of omni-directional binaural in-ear microphones (type 4101-B; Bruel and Kjaer, Virum, Denmark) was placed at the artificial ear canal entrances of the KEMAR manikin to record sine sweeps from each loudspeaker. The KEMAR HRTFs were created by deconvolving the sine sweeps from the binaural recordings.

**Fig. 1.**
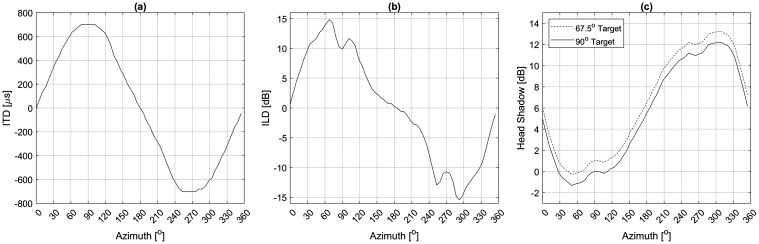
ITD and ILD as a function of azimuthal positions from the head-related transfer function.

Figure 1 shows the ITD calculated from the KEMAR HRTFs by taking the delay at the peak of the cross-correlation between the binaural signals after low-pass filtering at 1500 Hz, and the high-passed ILD at 2000 Hz as the root-mean-squared difference. In the frontal azimuth, ITD had a monotonic increase from 0° to 90° from 0 *μ*s to ∼750 *μ*s, whereas ILD reached a peak of ∼13 dB at 67.5° before reducing in magnitude until 90°. The head shadow quantified as the difference in target-to-masker SNR between ears is shown in Fig. 1(c) as a function of the masker position. With a target position at 67.5° or 90°, there is minimal head shadow (< 3 dB) available when the masker is located in the same hemi-field and beyond 10° azimuth.

During the experiment, listeners were seated in a sound booth and wore a pair of Sennheiser HD650 open-back headphones (Sennheiser GmbH & Co., Berlin, Germany). The spatial audio was created trial-by-trial by convolving the clean speech with HRTFs of the intended spatial location in a custom software made in matlab (v2021a; MathWorks, Natick, MA). For audio calibration in the experiment, a 60 dB sound pressure level (SPL) speech from the 0° azimuth in the virtual auditory space had a 60 dBA SPL in the left channel of the headphones as measured by a sound level meter.

### 2.3 Materials

Target speech was sentences with three keywords from the AuSTIN sentence corpus (Dawson *et al.*, 2013) recorded by a female talker speaking in American English. A two-talker speech babble was created from a second female talker speaking two distinct continuous discourses of science stories (Time for Kids, https://www.timeforkids.com/). In each continuous discourse, any silence gap longer than 100 ms was removed to minimize opportunities for glimpsing (Best *et al.*, 2017; Glyde *et al.*, 2013). During the experiment, the target speech was always presented at a fixed target position, either at +67.5° or +90° azimuth (on the listener's right) in the virtual auditory space (Peng and Litovsky, 2022). Depending on the trial, the two-talker masker may be co-located with the target or adaptively moving away toward 0° azimuth.

### 2.4 Procedure

Listeners were instructed to repeat back AuSTIN sentences. An experimenter sitting outside the sound booth scored the correct keywords from the listener on a trial-to-trial basis in the custom matlab software. A trial was coded as correct with two or three keywords responded correctly and incorrect with zero or one keywords. Accuracy was calculated based on keywords correctly identified.

The general procedure to measure MAS was similar to that described by Peng and Litovsky (2022) in two steps. First, we determined the SRT at 50% accuracy when the target and masker were co-located. To measure the co-located SRT, the masker level was fixed at 55 dB SPL; the target speech level was initially set at 60 dB SPL and adjusted using a one-down, one-up adaptive staircase procedure (Levitt, 1971). Two adaptive tracks were interleaved and each terminated after six reversals. A logistic regression was fitted to all trial data of keyword accuracy from each interleaved track using the “psignifit” toolbox (Frund *et al.*, 2011) to reconstruct the SRT psychometric function. The SRT was estimated as the SNR at the 50% point of the psychometric function. If the interleaved SRTs deviated from one another by >2 dB, the co-located SRT was re-measured for a more stable SRT. The lower (better) SRT from the two interleaved tracks was chosen as the SNR for 50% keyword accuracy for the MAS measurement.

In the second step, the MAS was measured by moving the two-talker masker together away from the target toward 0° azimuth to achieve 70.7% accuracy. For this study, three measurement methods were compared to understand their capability to expedite MAS measurement: (1) method of constant stimuli, (2) adaptive staircase, (3) and progressive tracking in both ascending and descending order. For the method of constant stimuli measurement, five angular separations were chosen with each repeated in ten sentences; all 50 sentences were presented in randomized order within a single run. For the adaptive measurement, MAS was measured using a single track of a two-down, one-up staircase terminated after six reversals and repeated in two runs. For the progressive measurements, two sentences (six keywords) were tested in each test angular separation with a step size of 11.25° with the two-talker masker either moving away (in ascending order) or moving toward (in descending order) the target. Each participant was assigned the order of testing MAS using the three methods in a pseudo-randomized manner following a Latin Square. When measuring MAS using the progressive method, the direction of masker displacement was counter-balanced: Half of the participants completed the ascending order before the descending order; the other half in the reversed order.

In addition to the four MAS measurements, a fifth MAS measurement was performed using the method of constant stimuli by setting the SNR as the SRT of 30% accuracy from Step 1. This allowed additional data to generate the three-dimensional psychometric functions relating the three parameters for spatial release from masking. To estimate the threshold for each MAS measurement, all trial data were included and converted into keyword-based percent correct and fitted with a logistic regression using the “psignifit” toolbox (Frund *et al.*, 2011), with MAS estimated at 70.7% keyword accuracy on the reconstructed psychometric function.

## 3. Results

All statistical analyses were performed using R (version 4.4.0) and RStudio (version 2023.12.1, Build 402), with packages including “buildmer” (version 2.11) “lme4” (version 1.1–35.3) and “boot” (version 1.3–30). To screen for any practice effect, Wilcoxon tests were performed comparing the medians between the first and second track of MAS using the adaptive staircase method. The distribution with individual data of MAS from two adaptive tracks had similar group-level medians when the target was positioned at 67.5° (*W* = 77, *p* = 0.15) and 90° (*W* = 58.5, *p =* 0.19). Due to the relatively small sample size, bootstrapping of the median was performed with 5000 repetitions and showed highly overlapping 95% confidence intervals (CI) across tracks and target positions (see supplementary material, figure Asupplementary material, figure A). With comparable thresholds, the adaptive MAS was averaged across the two tracks before the next comparison with other measurement methods.

**Fig. 2.**
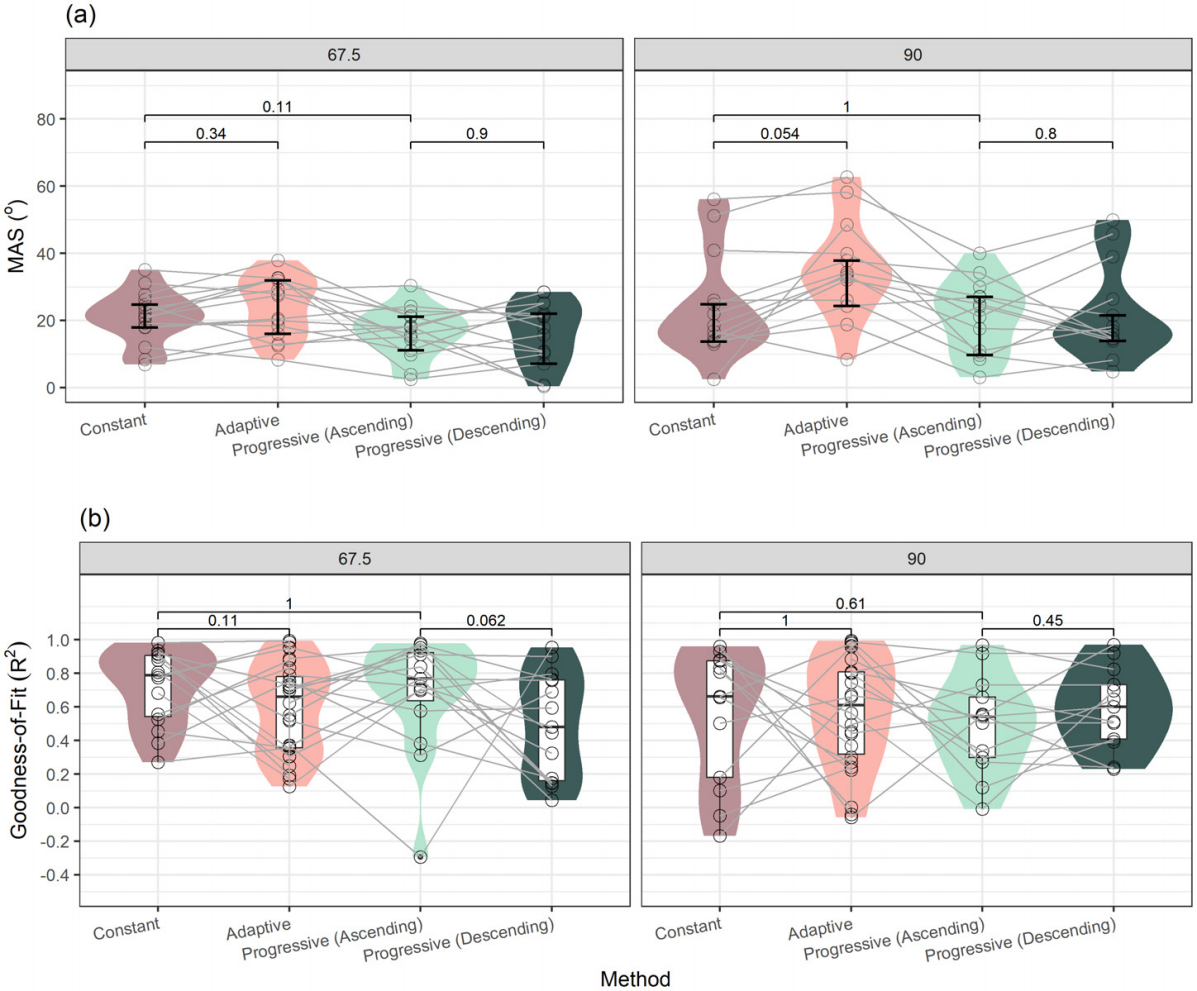
Violin plots showing distributions of (a) MAS and (b) goodness-of-fit from fitted psychometric functions as measured across four psychophysical methods: Method of constant stimuli, adaptive staircase, and progressive tracking in ascending order and descending order. MAS measured with target positioned at 67.5° (left panel) and 90° (right panel). Individual data overlaid on the violin plots. Error bars indicate 95% confidence intervals of the bootstrapped medians for MAS and boxplots showing medians and distribution percentiles of goodness-of-fit. All planned paired comparisons shown on figure, with unadjusted *p*-value listed. No significant paired comparisons detected at *p* < 0.05.

**Fig. 3.**
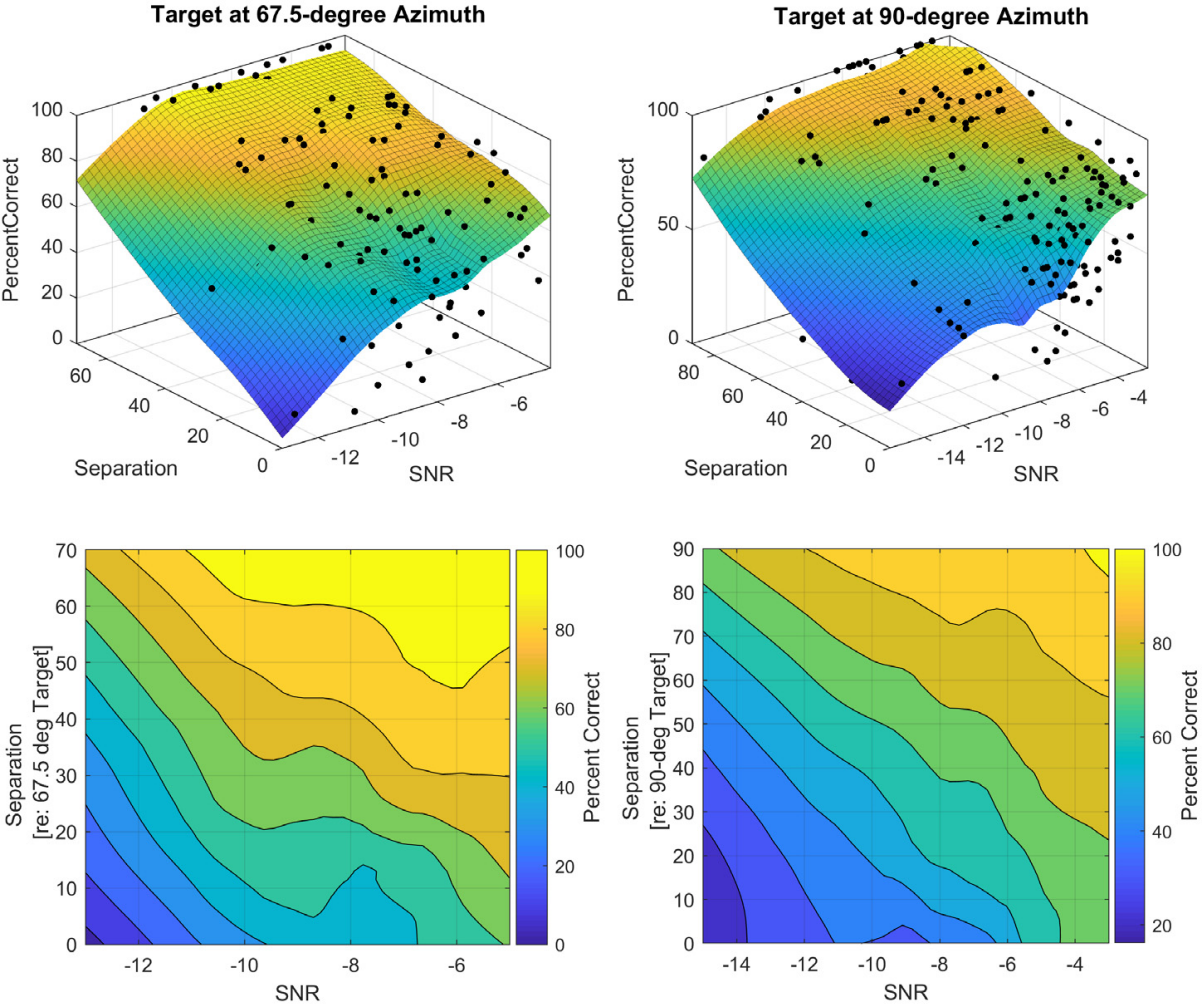
Accuracy as a function of target-masker separation and SNR for target at (A) 67.5° and (B) 90° azimuths. Surface fit to individual data tested using the method of constant stimuli (top row) and equal accuracy contour (bottom row) with color gradient indicating percent correct.

Figure 2 shows comparisons of MAS measured using different psychophysics methods and goodness-of-fit in $R^{2}$ of the fitted psychometric functions of each measurement. We split the progressive measurements by the direction order (ascending vs descending) to set up four levels of the method variable to allow further examination of direction bias in the *post hoc* analysis. A linear mixed effects model was fitted to the MAS thresholds with method, target position, test order, and their interactions as the fixed effects and participants as the random effect. An optimization procedure was applied to the initial model to reduce model dimensions using the “buildmer” package. The final model included method [*F*(3, 112) = 8.14, *p* < 0.001] and target position [*F*(1, 112) = 16.50, *p* = <0.001] as significant main effects, but excluded the main effect of test order. Participants remained in the final model as the random effect. On average, MAS measured from 90° target (*M* = 25.8°, SD = 14.8°) was larger by 8.3° than that from 67.5° target (*M* = 19.5°, SD = 8.9°). *Post hoc* analysis using planned paired comparisons with Wilcoxon tests was performed on the pairs of MAS thresholds shown in Fig. 3(a). Even though there was a significant main effect of method, the paired comparisons did not detect any significant difference between each pair of interest. No interaction was included in the final model. A similar analysis of paired comparisons was performed on the regression fit for the reconstructed psychometric functions. As seen in Fig. 3(b), none of the paired comparisons of interest was statistically significant at *p* < 0.05.

Next, we explored the relations between spatial separation, SNR, and percent accuracy, as illustrated in Fig. 3 with the three-dimensional surface fit. The surface fits were performed in matlab using the curve Fitter toolbox and Lowess fit type using a linear polynomial model with both SNR and spatial separation centered. The final surface fit models had an adjusted $R^{2}$ of 0.68 and 0.65, respectively, for the two target positions. Overall, the surface fit achieved moderate goodness of fit to represent the interrelation between the three parameters for spatial release from masking. The equal accuracy contours in Fig. 3 show how the relationship between accuracy (color gradient) and spatial separation (*y*-axis) varies with changing SNR. The trend of increasing accuracy with increasing target-masker separation and SNR is common between the surface fits from a 67.5° and 90° target, although accuracy reaches the ceiling sooner with a 67.5° target.

**Fig. 4.**
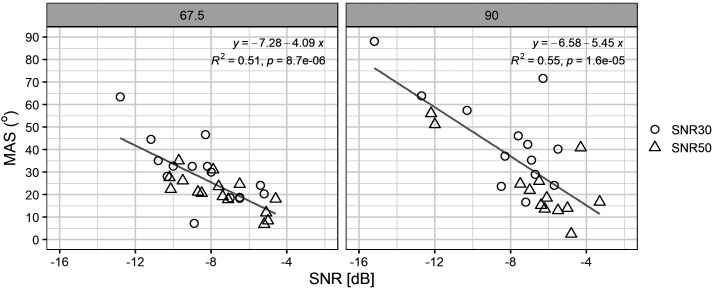
MAS as a function of SNRs at levels of 30% correct (circle) or 50% correct (triangle) when target and two-talker masker are co-located. MAS data measured from the target position at 67.5° (left panel) and at 90° (right panel) azimuths. Significant linear relations between SNR and MAS demonstrated by linear regression fit.

Figure 4 illustrates the significant and strong linear relationship between MAS threshold and SNR. For adults with typical hearing, every 1 dB increase in SNR is associated with an MAS reduction by 4.1° when the target was positioned at 67.5° and by 5.5° when the target was at 90°.

## 4. Discussion

The present study described a procedure to measure the MAS necessary to achieve a 20% intelligibility improvement in a sentence repetition task with a two-talker babble masker. The MAS threshold measurement includes two steps. First, the co-located SRT at 50% correct was measured. Second, by setting the SNR at the co-located SRT, the MAS was measured by moving the two-talker masker away from the target to achieve 70.7% correct. We compared MAS measured from the method of constant stimuli with those measured from the adaptive staircase and progressive tracking methods and found no significant difference in the threshold estimation across methods even after bootstrapping. Similar to Ahrens *et al.* (2020), using the adaptive staircase method, we found no practice effect or improvement by repeating MAS measurements. Interestingly, MAS measured from progressive tracking were comparable to those from the method of constant stimuli, both in the threshold estimation and in the psychometric function fit ( $R^{2})$. This finding was unexpected but encouraging for a faster procedure to reliably assess MAS, by reducing the measurement time by more than 50% to 14–18 sentences. Our finding also adds to the existing literature that validates the use of progressive tracking to expedite spatial unmasking measures of not only SRT (Jakien *et al.*, 2017) but also the spatial angle.

All listeners had measurable MAS < 90° with target and masker in the same hemi-field where minimal monaural head shadow cue <1 dB was available for unmasking [see Fig. 1(c)]. When the target was at 90°, as the masker moved away from the target toward 0° azimuth, listeners might experience a non-linear increase in ILD contrast between the two sound sources [Fig. 1(b)] available for unmasking: The ILD contrast first increased as the masker moved from 90° co-located with the target sharing the same ILDs until it reached a maximum at 67.5° azimuth, where the masker has the largest ILD), then decreasing ILD contrast from 67.5° as the masker ILD reduced until it arrived at ∼35° azimuth. When the target was positioned at 67.5°, the average MAS was 19.5°, corresponding to a difference in ILD of 2.7 dB and ITD of 135 *μ*s between the target and masker positions; and with the 90° target, the average MAS was 25.8°, corresponding to a difference in ILD of 4.5 dB and ITD of 59 *μ*s. The MAS in degree measured from two target positions were similar. However, depending on how much interaural differences were physically available, listeners seemed to be able to capitalize on the cue that provided better contrasts for speech understanding from the target-masker separation, whether it was ITD or ILD. This corroborates findings from previous work that NH adult listeners do not seem to prefer ITD or ILD in spatial unmasking as long as the cue is salient enough for sufficient contrast (Edmonds and Culling, 2005b,a).

In this work, we also explore the co-dependent relationship between target-masker spatial separation and SNR on accuracy (Fig. 3). When SNR is low, accuracy experiences a more gradual and linear increase with increasing spatial separation. When SNR is high, accuracy starts at a higher level and has a more rapid increase with increasing separation. We expand the evidence of a non-linear relationship among the three parameters involved in spatial unmasking measures (Srinivasan *et al.*, 2016).

In the context of release from masking, our current measure of MAS is similar to the minimum audible angle (MAA), the measure of spatial acuity to quantify the smallest spatial separation needed to discriminate two sound sources. Normal-hearing adults have high spatial acuity with an MAA of ∼2° around 0° azimuth region, but drastically larger >40° around 90° azimuth (Mills, 1958). Similarly, we anticipate that our current MAS with an off-center target reported here may be an underestimation with a target closer to 0° azimuth. Future work is warranted to examine the effect of target position on MAS.

In conclusion, our fast measure of MAS offers another way to assess spatial unmasking, with the ability to characterize individual access to interaural difference cues for spatial unmasking.

## Supplementary Materials

See supplementary material for data comparing two repeated measurements of MAS using the adaptive staircase method.

## Data Availability

The data that support the findings of this study are available on reasonable request from the corresponding author, Z.E.P.
